# HEALTHY RESIDENCE IN RICHARDSVILLE: A PHOTOVOICE PROJECT ON AGING IN PLACE

**DOI:** 10.1093/geroni/igz038.2957

**Published:** 2019-11-08

**Authors:** Terri Lewinson, Gaynell M Simpson

**Affiliations:** 1 Georgia State University, School of Social Work, Atlanta, Georgia, United States; 2 University of Alabama, Tuscaloosa, Alabama, United States

## Abstract

Based on AARP’s domains of community livability, this presentation presents findings from a photovoice study of residents in an urban community in Atlanta, Georgia. Richardsville (pseudonym) is one of many communities in Atlanta’s inner city that has transformed from a blighted, long-derelict area to a hotbed of high rent apartment units, mixed with quarter-million-dollar-plus homes. With property values skyrocketing and rents soaring, it has becoming increasingly difficult for longtime, older residents of this historically African-American community to remain in the vicinity. Richardsville Senior Residences, an affordable rental unit, was built to provide housing for a mix of incomes and ages and retain longtime residents in Atlanta’s neighborhoods. Therefore, from this qualitative study, we share about residents’ motivations for moving into Richardsville Senior Residences and their perceived livability of the environment that influence healthy aging in place.

